# Online Pre-Order Systems for School Lunches: Insights from a Cross-Sectional Study in Primary Schools

**DOI:** 10.3390/nu14050951

**Published:** 2022-02-23

**Authors:** Nahlah Alkhunain, Jennifer Bernadette Moore, Hannah Ensaff

**Affiliations:** 1 Nutritional Sciences and Epidemiology, School of Food Science and Nutrition, University of Leeds, Leeds LS2 9JT, UK; fsnka@leeds.ac.uk (N.A.); j.b.moore@leeds.ac.uk (J.B.M.); 2 Clinical Nutrition, College of Health and Rehabilitation Sciences, Princess Nourah Bint Abdulrahman University, Riyadh 11564, Saudi Arabia

**Keywords:** dietary habits, children, food environment, pre-order, parents, school nutrition, food choice

## Abstract

Schools are increasingly using online pre-order systems for children to select school meals in advance. This study aimed to explore how children use and interact with these systems. Using a combination of direct observation and an online questionnaire, the operation of these systems in four UK primary schools was examined. This included how the menu options were displayed, how these were selected by children (4–11 years), and the interactions between children and others when making food selections. Where possible, most children pre-ordered their school lunch in the classroom, and differences in the food choice process among children were observed. These apparently related to children’s ages; older children (8–11 years) showed more independence when making food selections, whereas younger children were often supported by others. Most parents reported that their child was the decision maker when pre-ordering the school lunch, and the role of children in the selection of school lunches was evident. This may be accentuated by the online pre-order systems, and given the likely expansion of these systems in schools, there is an opportunity to implement interventions to influence children towards specific or different meal options from the school menu.

## 1. Introduction

The prevalence of global childhood obesity has increased substantially in the last forty-five years [1]. In England, the prevalence of excess weight increases during the early school years, with the most recent data showing 28% of children (ages 4–5 years) starting school are overweight or obese, increasing to 41% by the final year of primary school (ages 10–11 years) [2]. For optimal health, children’s energy and nutrient intakes should fall within age-appropriate recommendations. Although parent-reported energy intakes for UK children (aged 4–10 years) are not too far off recommendations, the consumption of free sugars and saturated fats typically exceeds recommendations [3].

Excess weight gain in children has been reported to be associated with the intake of foods, such as potatoes cooked in oil (french fries, roast potatoes, crisps), processed meats, coated poultry and fish, milk, sugar-sweetened beverages, desserts, and sweets [4]. Both internal and external factors (e.g., hunger, personal likes, and food availability) can influence children’s food choices. Children themselves have reported that their parents are key sources of such drivers of food decisions, as well as their peers [5]. Understanding children’s food choices is a critical step in guiding better diets that meet dietary recommendations. Researchers have employed various methods to examine children’s food choices, and these have included digital images from cameras and smartphones [6,7], cashless catering data [8,9], and qualitative research with children [5,10], as well as recording food components on children’s lunch trays [11], and observing children’s selections in school [12]. Better understanding of food choice within a school setting is critical to informing the development of interventions and school food policy. Previous school-based interventions have highlighted the potential to change food choice, including towards healthier options [13], fruit [14], vegetables [15], and plant-based foods [16].

Several theoretical models are relevant to understanding children’s food choice behaviour. The Socio-ecological Model [17] describes the relationships among individuals, groups, and their environments and can provide insights into how pupils interact with others within the school environment when choosing their school lunch. This model has been used to examine health behaviour promotion [18], and promoting healthy dietary choices [19] in schools. Another model, the Food Choice Process Model, describes the factors and concepts involved in making food choice decisions [20], and can provide important insights into how individuals choose food. It has previously been used to examine children’s food choices and relevant social factors such as parental and peer influence [21].

The school environment provides important opportunities to improve children’s diet and health behaviours [22]. Food-based and nutrient-based standards became mandatory in primary schools in England in 2008 [23,24]. In 2013, after a review of school food provision, the Department for Education published The School Food Plan which sought to improve the food available in schools and to positively affect children’s food choices [25]. In September 2014, the Universal Infant Free School Meal (UIFSM) programme was introduced in England, whereby all pupils in reception and key stage 1 (i.e., Reception to Year 2, age 4–7 years) can receive a free school meal [26]. This is separate to the free school meal programme which provides a free school meal to pupils, depending on family income and receipt of benefits, e.g., income support. In 2015, revised school food standards aiming to promote good nutritional health were introduced for all schools in England [27]. These food-based standards stipulate the types of food to be on offer and how often, e.g., at least one portion of vegetables or salad as an accompaniment every day, at least one wholegrain variety of starchy food every week, oily fish every three weeks or more often, no more than two portions of deep-fried/batter-coated/breadcrumb-coated food each week [28]. There is a distinction to be made between the broad range of foods on offer for children to choose from and what children then select for their school lunch. This highlights the importance of a better understanding of how children choose food from school menus and the relevance to children’s dietary intake.

School lunches in England may be provided through local authority catering providers, in-house services, or private contracted companies. Schools generally operate a three-week menu cycle, with several meal options available every day, from which pupils make their selection. Primary schools are increasingly using online pre-order systems (OPSs) for pupils (and their parents) to choose their school lunches. A better understanding of these systems in operation will provide the necessary foundation for potential OPS-based interventions, to support children’s food decisions and, ultimately, to contribute towards improving dietary intake. This study aimed to explore how OPSs are used within a school food environment, and to examine how pupils interact with these systems to select their school lunches, and what this reveals regarding children’s food choices. 

## 2. Materials and Methods

This cross-sectional study was designed employing both direct observation and questionnaire survey methods. Ethical approval was granted by the University of Leeds Faculty Research Ethics Committee (ethics reference MEEC 18-017). 

All primary schools, within a local authority, that used OPSs were invited to participate in this study, i.e., schools that offered selection by an OPS of school lunch (defined as a meal provided at lunch time by the school). Schools were initially contacted by email, followed up by a telephone call and an initial school visit. Four out of the nine primary schools invited agreed to participate. These schools varied in size from the average size of a primary school in England (with approximately 280 pupils) to the largest with almost 500 pupils. The free school meal (FSM) profiles of the schools varied; three schools had a lower percentage of pupils eligible for free school meals than the national average for primary schools (21.6%) [29] and one school had a considerably higher percentage of pupils eligible for free school meals, at just over 30%. 

Reconnaissance visits were conducted at recruited schools, to understand how the schools managed school food and the lunch service, and specifically how the OPS was utilised. A drop-in session with parents was held at one school, to meet parents, answer questions, and informally collect data on parents’ thoughts on their children’s school lunch selections. Field notes from reconnaissance activities were written up by the researchers upon return, alongside reflections, and then discussed. Initial findings from these reconnaissance visits (e.g., how the OPS was utilised in the classroom, lunch service was managed) further informed the study design.

### 2.1. Direct Observation

For the first component (non-participant direct observation), pupils’ processes for selecting school lunches using the OPS, as well as their mealtimes in the dining areas, were observed directly and systematically. Direct observations can offer rich insights into what people do in public and capture people’s behaviours in different social contexts [30]. They provide an opportunity to record actual rather than reported behaviour, and are useful in understanding children’s behaviour, particularly in real-world settings. For example, this method has been used in schools to observe 9–10-year-old children eating school lunches and to record items and amounts eaten by children [31].

A bespoke observation form was developed to capture basic details and to note pupils’ apparent behaviours and experiences when selecting and consuming school lunches. The form was developed based on the aims of the observation, i.e., to capture: (1) how pupils chose their school lunches using the OPS; (2) how the school lunch options were displayed on the OPS; and (3) pupils’ interactions with others (e.g., peers, teachers, parents) within the school food environment. The design of the observation form was informed by previous research [32] and was checked and then refined following initial school visits and the drop-in session with parents; for example, details were added to how food was presented on the boards and to the availability of food in the dining area.

The final observation form was made up of two parts. First, observations in the classroom related to (a) pupils selecting a school lunch, e.g., number of pupils, when and where lunch was selected, i.e., during registration or outside school; (b) menu options on offer; (c) how pupils selected their school lunch, e.g., independently, with help from teachers or parents; and (d) the classroom environment, e.g., health promotion posters. Second, observations in the dining area related to (a) the availability and presentation of food; (b) interactions with catering staff, e.g., verbal prompts to pupils; and (c) dining area environment, e.g., information on school meals. The form was designed in a tick box style (wherever possible) to facilitate data collection, although some sections were ‘open’ to consider the potential heterogeneity of observations. During each observation session, the observation form was completed, while the researcher watched and listened. Clear and detailed descriptions of what had been observed were noted, and reflections on these notes and what had been observed were added as soon as possible after the session. Photographs of specific aspects (e.g., food options displayed on the interactive board) and the setting in general (e.g., food service area) were also taken during visits. The researchers strove for discretion, limited their interaction with staff and pupils, and positioned themselves in an inconspicuous location.

#### Observation Data Analysis

Following each observation visit, data were collated and observation notes written up. The researchers reviewed all data and highlighted key aspects and discussed emergent issues; points of discussion were written up and reviewed. The researchers also discussed potential sources of bias and the importance of reflexivity, paying attention to how their own experiences and characteristics might influence their conclusions. Furthermore, throughout the observation period, a daily reflection log that referred to the notes and photographs was kept. This regular process of data collection, note-taking, daily reflection, and discussion amongst the research team helped to provide valuable and robust insights from the observation.

### 2.2. Parent Questionnaire 

An online questionnaire was developed to collect data on parents’ perspectives on their children’s food choices in school, factors affecting these choices, and the OPS. Of particular interest was how the OPS was used, including potential factors influencing food choice, related to the OPS. 

Insights from the observation visits, informal discussions with catering staff and teachers, discussion among the research team, and previous research [33,34,35,36,37] informed the development of the questionnaire. Items were incorporated with adaptations as required from previous research relating to school lunches and packed lunches [33] and family food habits [34]. The final questionnaire included three core sections: school meals (packed lunches and school lunches), food habits and eating behaviours, and demographic characteristics. In addition to closed-ended questions, several open-ended questions were included to give parents the freedom and space to answer in detail, e.g., ‘Do you have any other comments to make about your child’s school lunches?’ A different version of the questionnaire was developed for each school, to take account of different systems and school-specific rules, e.g., pre-ordering online only available at school, alternating between a school lunch and packed lunch being ‘allowed’ by the school.

Attention was paid to the wording and language in the items and literacy difficulty levels were assessed for the questionnaire, to check for appropriate readability scores [38,39] (Flesch–Kincaid Grade Level: 4.2; Flesch Reading Ease: 74.7%). The questionnaire was reviewed for content validity by a panel of experts and stakeholders, including: a school governor, parent, local government school food advisor, headteacher, school business manager, school support staff. The questionnaire was reviewed as a digital document and also in its online test version; feedback was received individually from experts and stakeholders, either in written form or through informal interviews, depending upon individual preference. Based on feedback, changes relating to the structure and clarity were made. The questionnaire was tested with 29 parents, individually and in person. Parents were asked about the types of questions included, and their structure, clarity, and whether the questionnaire’s contents were comprehensible. The time required for completion was also checked. This contributed to checking the questionnaire’s suitability and feasibility.

The platform used to host the questionnaire was Online Surveys (formerly Bristol Online Surveys). All parents were invited to complete the questionnaire. Introductory information was sent home via the schools’ communication systems (e.g., email, app, Twitter, newsletter), with a hyperlink to the questionnaire. Leaflets (including a QR code to link to the questionnaire) were given to the schools for pupils to take home. In addition, researchers visited three schools at morning drop-off and afternoon pick-up times with a tablet device and approached parents at the school gates to invite them to complete the questionnaire. The questionnaire was live from November 2019 through February 2020.

#### Questionnaire Data Analysis

The data collected via the online questionnaire were downloaded and screened for missing values, inconsistencies, or anomalies. Statistical analysis was completed using SPSS statistical software (IBM SPSS Statistics, Version 26.0, 2019, IBM Corp, Armonk, NY, USA). Fisher’s exact tests were conducted to investigate associations between child characteristics (year group/key stage, gender) and outcomes related to school food choice (such as having a school lunch or a packed lunch, who the decision maker was). For all analyses relating to key stage, there were two groups; reception and key stage 1 were collapsed into one group (R & KS1, corresponding to younger children aged 4–7 years), with the remainder being key stage 2 (KS2, corresponding to older children aged 7–11 years). The Index of Multiple Deprivation (IMD) was derived from postcodes provided by participants [40,41].

For open-ended questions, parents’ responses were collated, reviewed, and analysed using a thematic approach. Coding was done manually and iteratively; codes and themes were considered and discussed and then the data were reconsidered until a final set of themes emerged that were agreed on by the research team as representative of the data.

## 3. Results

### 3.1. Findings from the Observation Visits

Observation visits were conducted from April through June 2019. Across all schools, 12 h of direct observation in classrooms were conducted, and 682 children (Reception–Year 6; ages 4–11 years) were observed during registration when selecting their school meals using the OPS (School A, 140 pupils; School B, 250 pupils; School C, not applicable as online selection at school not offered; and School D, 292 pupils). This corresponded to approximately half of pupils (46%) attending the four schools. In addition, 8 h of direct observation in the dining areas were conducted to observe pupils (Reception–Year 6) across the four recruited primary schools. 

Findings from the observation visits were grouped into three distinct categories: (1) the online pre-order systems; (2) children’s school lunch selection using the OPS; and (3) the school dining areas. Key findings are described below with respect to these categories, and include: the food options displayed on the boards and how they differed depending upon the OPS; differences in the food choice process between children of different ages including the influence of others within the school environment; and the influence of the catering staff in the dining area. 

#### 3.1.1. Online Pre-Order Systems (OPSs)

Meal options were displayed on the OPS and reflected the daily options for schools’ respective menu cycles. The schools operated a 3-week menu cycle. They offered daily options of: meat/fish-based meals, (e.g., chicken curry with rice, roast beef with mashed potatoes, fish and chips); vegetarian meals, (e.g., vegetarian pizza with chips, vegetarian sausage with mashed potatoes, vegetable grill with potato wedges); and jacket potatoes (served with baked beans, tuna, or cheese). School C also offered a fourth option of a sandwich (e.g., tuna, cheese, egg). Additionally, vegetable side dishes were served with the meat/fish-based meals and with the vegetarian meals, and each child chose one or two of the vegetables available that day (e.g., peas and cauliflower, carrots and broccoli). This set up for school lunches is common in primary schools in England. Likewise, many schools adjust provision according to the pupil body, e.g., offering a halal meal option where there are a substantial number of Muslim children—this was the case in Schools B and D. 

Pre-orders for school lunches using OPSs were done at school, through an interactive whiteboard or a tablet device during registration at the start of the school day. Pre-orders were also made outside school via a parent’s online account. Importantly, these systems enabled parents to pre-select meals for their children and to know their children’s choices. The presentation and implementation of the OPS differed across the four schools, and two different OPSs from two different companies were observed. Schools A, B, and C used the same OPS (referred here as System 1); School D used a different system (System 2) (Table 1).

In Schools A and B, parents and children could choose a school lunch remotely (outside school) in advance via an online account (with any orders placed at home not being able to be changed in the classroom) or alternatively, choose in the classroom during morning registration, which was the more popular option. In School C, the lunch choice could only be done remotely in advance. 

Where children pre-ordered during morning registration (Schools A and B), children chose their school lunch using an interactive whiteboard or a laptop/tablet device. The whiteboard had a list of children’s names on it, and when a child pressed on their name, the meal options appeared on the board for them to choose from. In some classes with younger children, one pupil stayed at the board and helped others choose their meals or would take a tablet device around the classroom and ask the pupils to choose their meals. The meals were shown by name, with different background colours based on meal type (e.g., green for vegetarian meal). Some meals had descriptive words (e.g., yummy vegetarian sausage) or detailed descriptions (e.g., layers of pasta, cheese sauce, vegetables, and tomatoes, topped with a golden layer of melted cheese), but there were no pictures or photographs of the meals. 

At lunch time in the dining area, two touchscreen monitors (one for pupils and one for catering staff) were used. Each child approached the ‘pupil’ touchscreen and selected their name to reveal their pre-selected school lunch option on both pupil and staff touchscreens. The ‘staff’ touchscreen was also used by catering staff to check which children had received or were yet to receive their meal, as well as any dietary requirements or allergies. Children who had not pre-ordered were also indicated; these children were able to choose an option during lunchtime from whatever food would be left. Observations in the dining areas highlighted how service could be reliant on the OPS. For example, longer queues and waiting time ensued when the touchscreen stopped working, or children found it difficult to find their name quickly on the small touchscreens.

School D used System 2 where children selected their meals from an interactive white board showing their photos and names, and when a child has selected a meal, their photo is colour-highlighted to denote their selection. Before lunchtime, each child is given a coloured wristband corresponding to their pre-selected meal option, e.g., red wristband denoted the meat/fish-based meal option. At lunch, catering staff recognised which meal to serve to each pupil from the wristbands.

#### 3.1.2. Children’s School Lunch Selection Using the OPS 

Overall, just over half (52%) of the children observed during the visits chose a school lunch (as opposed to packed lunches). Variations were apparent, however; for example, at School B, the majority of the younger children in reception and key stage 1 chose a school lunch (86%), whereas in KS2, less than half of the children chose school lunches (49%). This reflects the UIFSM provision, nevertheless, across all schools, for some of the children who qualify for free school meals, their parents opted for them to bring packed lunches as opposed to a school lunch. 

Most children (70%) who had a school lunch used a classroom whiteboard or tablet during morning registration to select their school lunch option (as opposed to the alternative of remote selection, outside school, with a parent account). Further, there was a higher number of remote selections for younger pupils (e.g., 38% in Reception) compared to other year groups. Across all schools, differences in how children chose a school lunch from the OPS were observed. Some children were fully independent when making food selections, (i.e., reading the meals on offer, deciding which they wanted and selecting) whereas other children received help from their parents or teachers. Generally, and as may be expected, the differences observed appeared to relate to children’s ages. More independent food selection was observed with older children in Years 4–6 (8–11 years), whereas younger children (4–8 years) were typically supported by their parents or teachers. Parental management of lunch selection was especially apparent in the Reception classes, where all the children chose their meals with a teacher’s or parent’s help. 

In some cases, parents were observed reading aloud specific options on offer that day and describing or explaining the menu to children. The extent of parental interaction over menu options varied, with the potential influence exemplified by one case when a child asked their parent, ‘What’s the green meal?’, the parent said, ‘It’s not very nice’. In another example, after a child chose a cheese sandwich, the parent responded, ‘But you had a cheese sandwich last night!’. In other cases, parents reminded their son or daughter that they were familiar with and ‘knew’ some options, to prompt them to choose specific meals, or provided encouragement such as ‘You’ll like that’. Whilst some parents engaged and involved their children in selecting the school lunch option, in contrast, some parents quickly read through the options available that day and chose on behalf of their children. The level of control exercised by parents varied, and in one instance, a parent changed the child’s meal choice on the OPS, but when the parent left the classroom, the child went back to the board and changed it back. Throughout all classes, there were a few instances of children needing help, for example, due to learning difficulties, or blurred screen on the whiteboard with unclear menu options. 

The relevance of peer influence was also evident during the observation. For example, school food selections were a topic of conversation among some children as they discussed what they wanted to eat and asked each other about their choices. Interestingly, across all classes, cases of peers influencing children’s choices were observed. This included, for example, children keen to see what their peers chose and allowing them to choose first or asking them about their choice, before then making theirs based on their friend’s (imitation of peers). In a minority of cases, children asked their friends to choose a meal for them if they were close to the board. 

#### 3.1.3. School Dining Areas 

In all schools, pupils entered the dining areas by year group (Reception to Year 6). Children received the school lunch that they had pre-ordered during registration that morning, or that had been pre-ordered remotely (outside school) by their parents or themselves. The school lunch included vegetable side dishes for pupils with a meat/fish-based/vegetarian meal. At all schools, there was also a separate salad bar where children could select and help themselves to salad items (e.g., cucumber, carrot, lettuce) and a separate table for desserts which held the one/two dessert options for the day (e.g., seasonal fruit crumble and custard, flapjack and apple wedges).

The importance of catering staff and in particular their interaction with children was apparent in the observations. Catering staff asked children about, for example, their choice of vegetable side dishes, and used verbal encouragement, such as ‘great choice’ or ‘good choice’. Sometimes, older children were asked about their portion size, e.g., if they would like more food or more sides. At the end of lunch, when pupils would bin their leftover food in the food waste bin, catering staff would, on occasion, encourage children to consume more of their lunch if they noticed that most was uneaten. 

### 3.2. Findings from the Parent Questionnaire 

A total of 125 parents completed the questionnaire in full, and Table 2 presents the demographic characteristics of these parents and their children. Most respondents were mothers (81%), and approximately a third (34%) were from households of two adults and two children. The highest percentage of participants (57%) were white British and more than two-fifths (46%) of parents had a university degree or equivalent. Based on the IMD, more than a fifth were living in the most deprived areas (22% in quintile 1), and more than half came from less deprived areas (51% in quintiles 4 and 5). The respondents were parents of similar numbers of boys and girls (52% boys and 48% girls) across all year groups, although Year 6 was the least represented (9% of children). More than a fifth of parents (23%) had children who required a special diet related to religion (e.g., halal food), reflecting the high percentage of the sample that were Asian/Asian British: Pakistani (21%).

#### 3.2.1. School Lunches or Packed Lunches

Most respondents (*n* = 81, 65%) reported their children always had a school lunch, with the remainder taking a packed lunch (a small minority had a combination of school lunch or packed lunch on different days). More younger children (R & KS1, ages 4–7 years) had school lunches than older children (KS2, ages 7–11 years), and to examine the relationship between the type of lunch and key stage, Fisher’s exact tests were conducted. These indicated that there was a significant moderate association between the type of lunch and key stage, (χ^2^ (2, *n*= 125) = 17.99, *p* < 0.001, Cramer’s V = 0.38); this reflects the Universal Infant Free School Meal programme for pupils in Reception and key stage 1. 

School lunches were viewed positively, with the overwhelming majority of parents (94%) reporting their children enjoying them, and more than three quarters of parents (76%) reporting their children to be happy with the amount of food. Parents also reported that they were happy themselves with their child’s school lunch (89%) and the variety of food on the school menu (78%). Two-thirds, however, would prefer the school lunch menu to include healthier options (67%). Ultimately however, most parents (91%) would not prefer their child to have a packed lunch instead. 

When asked about the reasons for their children having a school lunch, most parents thought it was ‘very important’ for their children to have a hot school lunch (62%), to have a healthy school lunch (58%), to have a freshly prepared school lunch (53%), and to have something different to eat each day (51%). Particularly for parents of younger children (R & KS1), value for money and a freshly prepared school lunch were important (93% and 74%, respectively; compared to KS2, 8% and 26%, respectively) with a significant association between responses and key stage (χ^2^ (3, *n* = 79) = 9.10, *p* = 0.026, Cramer’s V = 0.33 and χ^2^ (3, *n* = 79) = 6.92, *p* = 0.034, Cramer’s V = 0.30, respectively), with a moderate effect size.

#### 3.2.2. Children Choosing a School Lunch Option from the OPS

More than half of parents whose children had a school lunch (45 of 84, 54%) reported that their child chose their own school lunch option, and most of these parents preferred their children to do so (36 of 44, 82%—one did not answer). More children in Years 2 and 3 (67%, 100%, respectively) chose their own lunch option, compared to other year groups (range 33–50%), and there was a significant association between year group and who chose the lunch option from the school menu (χ^2^ (18, *n* = 84) = 29.73, *p* = 0.007, Cramer’s V = 0.33) with a moderate effect size. In Schools A and B where parents and children could choose a school meal option remotely via an online account (as well as by using the boards in the classroom), many parents (21 of 33, 64%) reported choosing meals remotely using a device (e.g., mobile). This was not evident in the observation where most children chose their school lunch at school.

#### 3.2.3. Different School Lunch Options from the School Menu

Regarding the school lunch options, more parents of R & KS1 children liked their children to eat a mix of meals (30%) or a meat/fish-based meal (13%) compared to parents of KS2 children (17% and 1%, respectively). More KS2 parents (13%) also did not mind about the option chosen compared to R & KS1 parents (4%). Overall, there was a significant association between key stage and parents’ preferred meal options, (χ^2^ (6, *n* = 84) = 15.10, *p* = 0.008, Cramer’s V = 0.42) with a medium effect size.

**Figure 1:**
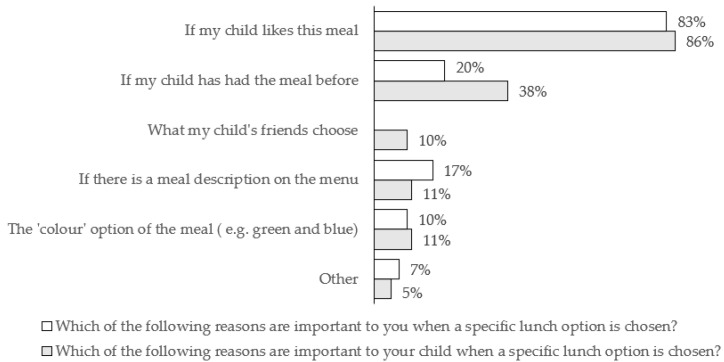
Parents’ perspectives on reasons (for themselves and for their children, separately) that a specific lunch option is chosen, *n* = 84.

Parents were also asked about the reasons that a specific lunch option is chosen (Figure 1). Most parents reported their child liking the meal was important for them (83%) and also important for their children (86%). Likewise, familiarity (i.e., ‘If my child has had the meal before’) was important to children (38%) and to parents (20%). In particular, familiarity was important to more R & KS1 parents (14%) than KS2 parents (6%), and reported to be important to more R & KS1 children (24%) than KS2 children (14%). A significant association between child’s year group and the importance of familiarity was observed (χ^2^ (6, *n* = 84) = 13.55, *p* = 0.027, Cramer’s V = 0.42). A minority of R & KS1 parents (10%) reported that ‘What my child’s friends choose’ is important to their children (no KS2 parents chose this reason), and a significant association with key stage was observed with a small effect size (χ^2^ (1, *n* = 84) = 5.72, *p* = 0.020, Cramer’s V = 0.26). 

**Figure 2:**
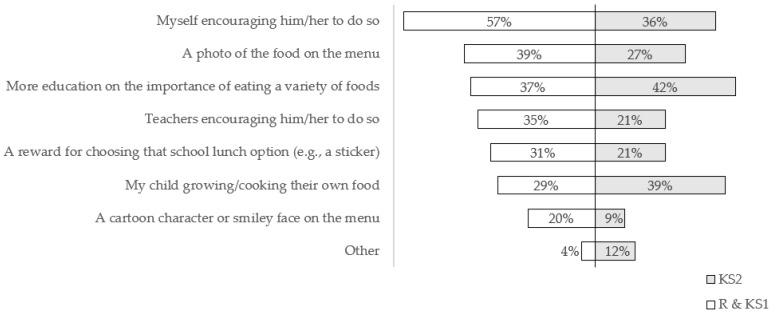
Parents’ perspectives on what might help their children choose a different school lunch option from the school menu, *n* = 84, % of parents of younger children (R & KS1; ages 4–7 years) and % of parents of older children (KS2; ages 7–11 years), separately.

Parents were asked about what might help their children to choose a different school lunch option from the school menu. Almost half (49%) of all parents felt that encouragement from themselves, more education on the importance of eating a variety of foods (40%), a photo of the food on the menu (35%), and children growing/cooking their own food were important (34%). Figure 2 provides the key results for R & KS1 and KS2 parents, separately. It is interesting to note that there are two distinctions, with education on the importance of eating a variety of foods and children growing/cooking their own foods featuring more for parents of older children. Specifically, parents of younger children (29%) thought their children growing/cooking their own food might help their children to choose a different school lunch option compared to parents of older children (39%), and there was a significant moderate association between child’s year group and whether parents thought that growing/cooking their own food might help (χ^2^ (6, *n* = 84) =12.89, *p* = 0.034, Cramer’s V = 0.40).

#### 3.2.4. Emergent Themes from Parents’ Comments

There were 151 free text comments made by parents in response to open-ended questions. Analysis of the comments revealed three main themes, with component subthemes (Table 3).

**Wanting children to eat better**

While a few parents were ‘happy with my child’s diet’, many referred to wanting their children to ‘eat better’ or ‘eat healthier’. Specifically, parents commented about wanting their children to have a greater variety of foods, to try new foods (particularly fruit and vegetables), and to consume less sugar.

*‘I want my son to have a more varied diet. I would like to be able to offer a more interesting range of things to my child! He eats nothing!’*

(Reception parent)

A few parents referred to their children as being ‘fussy’ or ‘picky eaters’, and wanting them ‘not to be so fussy and try new things’. Parents generally referred to wanting their children to try new foods and be ‘more adventurous’, particularly with fruit and vegetables, which was also highlighted by a few parents as a food group that they would like their children ‘to try a broader range of’.

*‘A greater variety of vegetable and willing to eat or try new things so the whole family could eat a more varied diet’*

(Reception parent)

*‘More fruit and vegetable, and would like him to have a more adventurous palate’*

(Year 1 parent)

*‘I would love for my son to trust food more and be less cautious. I wish my child would try new things’*

(Reception parent)

Some parents also referred to wanting their children to ‘*reduce sugary foods such as Apple juice*’ (Year 6 parent) in relation to improving children’s diets.

**Children choosing school lunch**

Overall, almost a quarter of comments related to children choosing their lunch and were positive, for example, relating to being able to ‘*Trust children to choose their school lunch*’. The most striking comments related to giving children independence or control when choosing their school lunches.

*‘I think it’s important for them to be independent and she [daughter] always chooses different things to eat’*

(Reception parent)

Other parents mentioned that their children preferred to choose by themselves from the school menu.

*‘He [son] likes when he is given (a) few options and he can choose his favourite’*

(Year 2 parent)

Parents also confirmed the value in their children choosing the school lunch from the school menu.

*‘He [son] knows what he likes better than I do. If I chose for him, he would not eat it’*

(Year 3 parent)

**Improving the school lunch**

Wanting improvements to the school lunch was also noted, this included more varied options, the ‘quality’, and portion size. Some parents commented on adding more varied options to the school menu.

*‘I would like to see a wider range of foods as the same meals appear on the menu too often’*

(Year 6 parent)

Parents also suggested improving the quality of the school lunch and mentioned that vegetables can be overcooked ‘soggy’ or ‘like mush’.

*‘The quality is pretty poor, for instance over cooked vegetables’*

(Year 3 parent)

Other parents referred to a need to increase school lunch portion sizes as their children are hungry when they arrive home from school.

*‘Sometimes the portions are a bit small so he can come home very hungry!’*

(Year 3 parent)

*‘The portion of food may not be enough for most of the children as they are seemed to be starving after school’*

(Reception parent)

## 4. Discussion

This study revealed insights into online pre-order systems in primary schools, specifically, how pupils interacted with others when choosing their school lunch, differences in the food choice process, as well as how menu options were displayed. The analyses also highlighted parents’ perspectives on their children as decision makers when it came to choosing the school lunches–with many parents preferring this. Further, comments from parents cited children’s independence when choosing school lunches as a part of the independence of growing older. Findings from the observation indicated that most children chose their school lunch during morning registration. The role of children in food decisions corresponds with previous research, which has highlighted children’s influence on food at home [42,43,44], food outside the home [45], as well as packed lunches in school [10].

In this study, distinct differences between older and younger children were found. This related to how children themselves were observed to select their school lunches, as well as parents’ perspectives of their children. For example, younger children tended to need support in choosing their lunches; this is as might be expected and consistent with, for example, their younger age and relative literacy skills. Their reliance on others, however, introduced the potential influence of their parents or teachers. There also appeared to be a continuum of parental support and control (e.g., ranging from none where children made choices independently, to where parents selected the school lunch). More parents of KS2 children reported not minding which school lunch option their child chose compared to parents with younger children, pointing to a loosening of parental control over food choice with age. Likewise, whilst only a minority of pupils had lunches that had been selected remotely (outside school), a greater number of remote selections was evident for younger pupils—this may be a reflection of parents’ greater input/level of responsibility. A previous study on the perspectives of parents and children (5–12 years) found that, generally, with out of home food choice, children exerted the most control over the final food and that parental input depended upon the child’s age, with younger children being given less control to choose their food [45]. Other work has also found that younger children (7 years) appeared to have less control than older children (9 years) in food decisions at home and school [46]. This also supports our observation of the independence of older children compared to younger children when choosing their school lunches in morning registration.

In the present study, parents with younger children held whether their children’s friends chose a meal as an important factor for their children choosing a specific lunch option. This supported findings from the observation where incidences of peers influencing children’s choices were evident. This concurs with previous Australian research which found that parents considered peers to be an important influence on children’s food preferences and intake [47]. Likewise, previous research [5] found that parents and peers were seen as sources influencing drivers of food choices such as health, availability, and likes. 

The influence of parents and peers and the related social aspects are relevant to the Socio-ecological Model [17], along with other more distal levels of influence, such as the use of an online pre-order system (OPS) by the catering provider, and any related school rules (e.g., pre-selection only at home or only at school). Likewise, in this study, personal food preferences, and personal food system factors [20], were reported as the main reason that parents and their children chose a specific lunch option. This adds to previous research highlighting the importance of food preferences as a major influence on children’s food choices [48].

The direct observation of children’s food selections was useful in capturing children’s behaviour in the school environment. As well as providing a basic understanding of the practical aspects of how children are interacting with the OPS, it also provided insights into how children choose what to eat at school, and the potential of others affecting their choices within the school environment. This study is unique in that it observed the pre-ordering within a school environment, and also demonstrates the utility of the observational method for examining children’s food choices and environmental effects that influence them. Previous research has captured children’s food selections at schools by recording the food components on children’s trays [11], and fruit selections [12]. It is also interesting to note that OPSs generate automatically collected food choice data. What is known about children’s food selections and nutrition interventions at schools is largely based on studies that observed children’s school meals and selections; OPSs provide a distinct opportunity to examine children’s food selections in primary schools, and similar datasets from secondary schools have provided useful insights [8,9]. 

With OPSs, particularly with pre-selection at school, it is proposed that children themselves are likely to be making their choices. This highlights the importance of the presentation of options on the digital screens, and also the relevance of others influencing them (e.g., teachers, parents, peers). Indeed, the results of the observation indicated that children interacted with the OPS and the offering of school meals on the boards. The extent to which the OPS influenced the selection by children, however, is unclear. Given the relevance of the framing of food choice, factors related to how the food options are presented (e.g., images of food, meal names, and descriptions), i.e., the choice architecture [49], within the OPS are important. This study provides valuable preliminary data, and findings point to the development of interventions to improve children’s meal choices, by, for example, adjusting the menu presentation, wording, order of options, and adding images, i.e., changing the choice architecture. These changes could promote the selection of certain food items, and interventions could aim to promote target foods on the basis of different aspects, such as nutritional composition, dietary diversity, or environmental sustainability. A previous study of an online ordering system intervention entailed behaviour strategies that included menu labelling, food availability, food placement, and prompting to improve healthy school lunches at ten primary schools (*n* = 2714 children, aged 5–12 years) and found these to be effective in improving food selection [13]. Another school-based study explored an intervention entailing the positioning of fruit and vegetable items as the first and final items on a menu in an online ordering system, to encourage their selection via online school environments, and recommended the use of strong intervention strategies, and more comprehensive behaviour interventions [50].

It is clear that there are opportunities with OPSs and, more generally, with pre-ordering school lunches. Potential disadvantages, however, should not be overlooked. A previous study on UK employees who used a pre-ordering website to select their lunches found that participants valued seeing the foods before selecting and some reported that not being able to view the foods on offer was a weakness of pre-ordering [51]. It is unclear whether this was the case with the OPS in schools, and further research to examine this would be worthwhile. 

Limitations of this study should be acknowledged, including the limited number of schools, all within the same local authority. The observation form was specifically developed for this study, however, it may not have captured all pertinent information. Likewise, data collected during observation were limited and subject to interactions happening around the researcher. Additionally, although researchers strived for discretion, the researchers’ presence may have affected how pupils chose their school lunches. Parents self-selected for the questionnaire and sample bias should be acknowledged as a limitation, as well as the relatively low number of responses. When considering findings, attention should be paid to the schools’ characteristics (including e.g., the FSM profiles, parents’ ethnicity), and further studies to examine other schools (including those with pupils from different socio-economic backgrounds) would be valuable, especially given the anticipated rise in the use of OPSs in primary schools. 

## 5. Conclusions

Pupils using an online pre-order system were observed to be the primary decision makers when choosing their options for school meals. Differences in levels of external control and influence were evident and these generally varied according to age. Online pre-order systems offer the potential of intervention, including targeted and age-differentiated strategies, to influence food choice and promote specific or different meal options from the school menu. They also provide valuable food choice datasets for the exploration of children’s meal choices.

## Figures and Tables

**Table 1:** Direct observation at four primary schools offering school lunch selections by online pre-order systems (OPSs).

School	Children (*n*)	School Lunch Selection	Meal Options on OPS	Lunchtime Service
A	140	System 1 Pupils selected their school lunch at school using an interactive whiteboard/tablet *or* Pupils/parents selected remotely using an online parent account	Three/four meal options Background colours according to meal type Some meal descriptions No images of meals	Pupils selected their name on the ‘pupil’ touchscreen monitor to reveal their pre-selected meal on the ‘pupil’ and ‘staff’ screen for catering staff
B	250			
C	228	System 1 Pupils/parents selected the school lunch remotely using an online parent account	n/a (no selection at school)	
D	292	System 2 Pupils selected their school lunch at school using an interactive whiteboard/tablet	Four meal options Highlighted colour according to meal type Background images related to meal options	Pupils wore coloured wristbands (according to their pre-selected meal option) to indicate meals to catering staff

**Table 2:** Demographic characteristics of respondents (*n* = 125) and their children.

Characteristic ^1^	*n* (%)
Parent’s Gender	
Male	23 (19%)
Female	100 (81%)
Parent’s Education	
University degree or equivalent	54 (46%)
Postgraduate degree	25 (21%)
GCSE/O Levels/CSE	20 (17%)
A Levels or Level 3 equivalent	14 (12%)
Other	5 (4%)
Parent’s Ethnicity	
White British	69 (57%)
Asian/Asian British: Pakistani	26 (21%)
Asian/Asian British: Indian	8 (7%)
Prefer not to say	3 (2%)
Other	16 (13%)
Household	
Two adults–one child	22 (19%)
Two adults–two children	40 (34%)
Two adults–three children	15 (13%)
Two adults–four children	6 (5%)
Three adults–one child	7 (6%)
One adult–two children	7 (6%)
Other	21 (18%)
Index of Multiple Deprivation	
Quintile 1 (most deprived)	22 (22%)
Quintile 2	13 (13%)
Quintile 3	13 (13%)
Quintile 4	44 (44%)
Quintile 5 (least deprived)	7 (7%)
Child’s Year Group (age)	
Reception (4–5 years)	21 (17%)
1 (5–6 years)	17 (14%)
2 (6–7 years)	22 (18%)
3 (7–8 years)	21 (17%)
4 (8–9 years)	16 (13%)
5 (9–10 years)	17 (14%)
6 (10–11 years)	11 (9%)
Child’s Gender	
Male	65 (52%)
Female	59 (48%)
Child’s Dietary Requirements	
Religious (e.g., halal food)	29 (23%)
Vegetarian or vegan diet	8 (6%)
Allergy/food sensitivity	7 (6%)
Other	5 (4%)

Abbreviations: GCSE, General Certificate of Secondary Education; O Level, Ordinary Level; CSE, Certificate of Secondary Education; A level, Advanced Level. ^1^ Not all participants provided all information.

**Table 3:** Emerging themes and subthemes from analysis of parents’ comments.

Theme	Subtheme
Wanting children to eat better	More variety of foods Trying new foods More fruit and vegetables Less sugar
Children choosing school lunch	Independence/control over choice Children liking to choose their school lunch Children knowing what they like/will eat
Improving the school lunch	More options Higher quality Larger portion size
